# Supplementary material for: PRAME induces genomic instability in uveal melanoma
Source: Res Sq. 2023 Apr 26:rs.3.rs-2861359. Preprint. [Version 1] doi: 10.21203/rs.3.rs-2861359/v1 (PMC10168463; doi:10.21203/rs.3.rs-2861359/v1)
Supplement: Supplement 1 [file NIHPPRS2861359V1-supplement-1.pdf]

Supplementary Files

This is a list of supplementary files associated with this preprint. Click to download.

- SuppTable1KaryotypeAnalysis.xlsx
- SuppTable2ProteomicsPRAME.xlsx
- SuppTable3ubiquitome.xlsx
- SuppTable4BioIDPRAMEMEL290all.xlsx
- SuppTable5coIPSMC1A.xlsx
- SupplementaryInformation.pdf
